# DNA repair mechanisms and *Toxoplasma gondii* infection

**DOI:** 10.1007/s00203-013-0944-0

**Published:** 2013-12-14

**Authors:** Beata Smolarz, Jan Wilczyński, Dorota Nowakowska

**Affiliations:** Department of Fetal-Maternal Medicine and Gynecology, Polish Mother’s Memorial Hospital Research Institute, 281/289 Rzgowska Street, 93-338 Lodz, Poland

**Keywords:** *Toxoplasma gondii*, DNA repair mechanism, Immune system

## Abstract

Lately, we can observe significant progress in understanding mechanism of DNA repair owing to fast methods of DNA sequence analysis from different organisms the revealing of structure and function of DNA repair proteins in prokaryota and eukaryota. The protozoan parasites survival depends on DNA repair systems. Better understanding of DNA repair systems can help in new antipathogen drug development. This review is aimed at updating our current knowledge of the various repair pathways by providing an overview of DNA repair genes regarding *Toxoplasma gondii* infections and the corresponding proteins, participating either directly in DNA repair, or in checkpoint control and signaling of DNA damage.

## Introduction

Some reports document the *Toxoplasma gondii* infection as related to extensive manipulation of host cell processes, including the control of the cell cycle, apoptosis and DNA damage response (Blader and Saeij 2009). In addition, pathogen may need to evade the host immune system (Blader and Saeij 2009).

The achievements in parasites infection are still not sufficient compared to the problem scale. The molecular genetics technologies have been instrumental in increasing our understanding of *T. gondii* replication within its host cell, DNA damages development and DNA repair mechanisms.

DNA sequencing analysis is a key tool in many fields. A large number of different sciences are receiving the benefits of these techniques, ranging from genetics, biotechnology and molecular biology. DNA sequencing is promoting new discoveries that are revolutionizing the conceptual foundations of many fields.

DNA sequence analysis in samples from different organisms (yeast, plant, bacteria, parasites and human) by methods such as the Sanger technique, the Maxam & Gilbert technique and the method of single-molecule sequencing with exonuclease has been proposed as a useful tool for the revealing of structure and function of genes encoding DNA repair proteins (Jazayeri and Jackson 2002; Wood et al. 2005; Martins-Pinheiro et al. 2007; Gill and Fast 2007; Singh et al. 2010; Lluch-Senar et al. 2013). Some of sequence technologies are methods based on atomic force microscopy, on the use of nanopores or ion channels and DNA microarrays (Mikheikin et al. 2006; Kozarewa and Turner 2011; Li et al. 2013). These technologies and approaches have been very important in increasing our understanding of DNA repair gene variability in various organisms, including parasite such as *T. gondii* (Brown and Blader 2009).

The past decade has seen important developments in the molecular tools to study *T. gondii* (Cleary et al. 2002; Chaussabel et al. 2003; Bradley et al. 2005; Hitziger et al. 2005; Saeij et al. 2005, 2007; Nowakowska et al. 2006a, b; Dellacasa-Lindberg et al. 2007; Frankel et al. 2007; Nelson et al. 2008; Xia et al. 2008; Gubbels et al. 2008).

Molecular analysis includes the development of transfection (Soldati and Boothroyd 1993; Donald and Roos 1993; Sibley et al. 1994) and proteomic technologies, sequencing of both host and parasite genomes (Blader et al. 2001; Saeij et al. 2007; Xia et al. 2008) and large-scale mutagenesis-based screens (Frankel et al. 2007; Gubbels et al. 2008).

In this review, we will focus our discussion on how the function of DNA repair mechanism contributed to the developments infections of parasites such as *Toxoplasma gondii*.

## DNA repair mechanisms and *Toxoplasma gondii* infection

*Toxoplasma gondii* belongs to the eukaryotic phylum *Apicomplexa*, which infects about one-third world population (Sullivan and Jeffers 2012; Nowakowska et al. 2013).

DNA repair system is very important for the survival of obligate intracellular parasite *T. gondii*. Recent genetic and bioinformatics analyses confirm the presence of DNA repair machinery in this lower eukaryote (Silva et al. 2007; Onyangoa et al. 2011; Achanta et al. 2012). However, little is known about DNA repair mechanisms and the proteins involved in apicomplexan parasites such as *T. gondii*.

Although various organisms (human, yeast, plant, bacteria and parasites) contain different DNA repair mechanisms, the repair of a specific DNA damage is often conserved from bacteria to human, and in many cases, the proteins are highly similar (Cromie et al. 2001). Experimental analyses indicate that a *T. gondii* DNA repair protein TgDRE (Tg DNA repair enzyme) belongs to a large family of proteins containing RNA recognition motifs (RRM), glycine-rich motifs (G-patch) and a specific motif named SF45. SF45 motif is similar to the human splicing factor 45 protein, which is a component of the spliceosome (Neubauer et al. 1998). The presence of the two first motifs RRM and G-patch suggests that TgDRE may also be involved in RNA metabolism in addition to its DNA repair activity. TgDRE may be essential for parasite growth as expected for a protein involved in DNA repair and conservation of genome integrity. Further analysis is now in progress to identify the protein partners of TgDRE in the *Toxoplasma gondii*.

The protozoan parasites survival depends on DNA repair systems that constantly supervise chromosomes to correct damaged nucleotides generated by cytotoxic agents, host immune pressure or cellular processes.

Pathogen entry into the cell is sufficient to cause the specific breaks. Resistance to antiprotozoan agents that induce DNA damage has been associated with increased expression of DNA repair genes and the development of *T. gondii* infections.

Among all DNA damages such as oxidation of bases, alkylation of bases, hydrolysis of bases, bulky adduct formation, mismatch of bases and double-strand breaks (DSB) are most mortal to cell.

Unrepaired DNA damage can lead to mutation, various diseases development or cell death. There are known five systems of DNA repair: pathway of direct reversion of damage, base excision repair (BER), nucleotide excision repair (NER), mismatch repair (MMR), homologous recombination (HR) and nonhomologous DNA end-joining (NHEJ).

Limited work indicates that BER undergoes parasites-related changes, which are likely to contribute to the accumulation of oxidative DNA lesions and mutations (Nathan and Shiloh 2000; Wilson and Bohr 2007; Almeida and Sobol 2007; Vonlaufen et al. 2008). The protozoan infection to cell is associated with reactive oxygen species (ROS) production (Vonlaufen et al. 2008). Immune system activation in response to parasite infections produces ROS (Finkel and Holbrook 2000; Halliwell and Gutteridge 2007). It is knowledge that ROS induce cellular proteins, lipids and DNA damage (Splettstoesser and Schuff-Werner 2002).

Base excision repair (BER) is critically important for repairing base damage induced by ROS. BER corrects small DNA alterations that do not distort the overall structure of DNA helix, such as oxidized bases, or incorporation of uracil. BER is classified into two subpathways: short-patch BER, a mechanism whereby only 1 nucleotide is replaced or long-patch BER, a mechanism whereby 2–13 nucleotides are replaced. BER is initiated by DNA glycosylases, which cleave *N*-glycosylic bond of damaged bases leaving apurinic/apyrimidinic site (AP site) (Wilson and Bohr 2007; Almeida and Sobol 2007). If not repaired by AP endonucleases in dividing cells, AP sites have dramatic consequences as they can lead to single and subsequently double DNA strand breaks, which are lethal to the cell (Evans et al. 2000).

Finally, it is postulated that processes similar to DNA BER must exist to rectify spontaneous and host-mediated damage in *T. gondii*.

It is knowledge that intracellular protozoan parasites are exposed to oxidative stress produced by immune effector cells (Nathan and Shiloh 2000; Vonlaufen et al. 2008). It has been established that parasites are highly sensitive to ROS (Vonlaufen et al. 2008). Therefore, intracellular organisms may suffer extensive damage to DNA that must be repaired for genomic stability and survival (Vonlaufen et al. 2008).

Very important to *T. gondii* is the generation of abasic sites in DNA, which result from removal of oxidized or alkylated bases. These sites are repaired by AP endonucleases during BER. AP endonucleases have been studied in kinetoplastid parasites (Perez et al. 1999).

Onyangoa et al. (2011) showed that AP endonucleases in the obligate intracellular parasite *Toxoplasma gondii* would be critical for viability by protecting against DNA damage and particularly in the context of host immune insults on the parasite. *T. gondii* possesses two apurinic/apyrimidinic (AP) endonucleases that function in DNA BER. Several study confirms the presence of two apurinic/apyrimidinic (AP) endonucleases in *Toxoplasma gondii*, the Mg^2+^-dependent TgAPE and Mg^2+^-independent TgAPN (Onyangoa et al. 2011). *Toxoplasma* TgAPE exists as two forms, the shorter one being in the cytoplasm and possibly the apicoplast. It is known that localization of TgAPE to the apicoplast is essential for *Toxoplasma* viability (Fichera and Roos 1997). Alteration of TgAPN levels alters susceptibility of the parasite to DNA damage. Overexpression of TgAPE failed to provide protection against alkylating agents. Further molecular biology study into the precise role of both (AP) endonucleases, TgAPN and TgAPE, in the parasite is an important future analysis.

In conclusion, DNA BER is essential for *T. gondii* viability but little is known about BER type of DNA repair machineries that exist in this parasite.

Nucleotide excision repair system removes short DNA oligonucleotides containing a damaged base (Hanawalt 2002). NER recognizes bulky lesions caused by carcinogenic compounds and covalent linkages between adjacent pyrimidines resulting from UV exposure. NER is further classified into global genome repair (GG–NER) that occurs everywhere in the genome, and transcription-coupled repair (TCR), which removes lesions in the transcribed strand of active genes. NER is a multistep process involving multiple proteins such as ERCC3, PCNA, RPA, XPA and p53 (Christiansen et al. 2000; Goukassian et al. 2000).

Most genes involved in nucleotide excision repair system are represented in eukaryotic parasites such as *E.* *histolytica*, *G. lamblia*, *P. falciparum* and *T. vaginalis* genomes suggesting that this mechanism could be potentially active in these parasites (López-Camarillo et al. 2009). Nucleotide excision repair system is an important repair pathway during the parasite *Schistosoma mansoni* life cycle (Silva et al. 2007), but the role of NER in *T. gondii* life cycle has not yet been clarified. The DNA damage in intracellular parasites is great, but very little is known about the NER type of DNA repair machineries that exist in *Toxoplasma*. Future work will be needed to further our understanding of the underlying nucleotide excision repair mechanism in *Toxoplasma*.

The mismatch repair (MMR) system is important for promoting the genetic stability of eukaryotes and prokaryotes (Martin and Scharff 2002). MMR proteins recognize DNA mismatches that are produced during DNA replication, homologous recombination, deamination of 5-methyl-cytosine or other forms of DNA damage. The repair process is then mobilized to correct these mutations. In addition, the MMR system also inhibits homologous recombination between nonidentical sequences. The MMR system is also thought to play a role in repair of oxidative damage by mechanisms that are not well understood (Skinner and Turker 2005). MMR is essential for maintenance of repeated sequences, as mutations in MMR genes are associated with a substantial destabilization of microsatellites (Karran 1996), and microsatellite instability increases with aging in humans (Ben Yehuda et al. 2000; Krichevsky et al. 2004; Neri et al. 2005).

MMR is a highly conserved repair pathway that functions in improving replication fidelity by correcting replication-associated base–base and insertion/deletion mispairs (Li 2008). MMR also suppresses HR and plays a role in DNA damage signaling (Li 2008).

The main MMR pathway is initiated by the recognition of a mismatch by the heterodimer consisting of the MSH2 and MSH6 proteins (also called MutSα). MutSα is responsible for the recognition of base mismatches and insertion/deletion (IDLs) in mono- to tetranucleotide repeats. This complex, MutSα, is able to recognize most base–base mismatches and short IDLs (Hsieh and Yamane 2008).

MutS homologues are very important components of the eukaryotic mismatch repair system. Moreover to repair mismatched DNA, mismatch repair enzymes are known in higher eukaryotes to directly signal cell cycle arrest and apoptosis in response to DNA-damaging agents. *H. sapiens* and many other organisms such as parasites including *T. gondii* have almost all MMR genes, incorporating the components of the MutSα (MSH2/MSH6) heterodimer, which strongly suggest that MMR could be an active DNA repair pathway. The consequences of defective mismatch repair (MMR) are mutagenic parasites that generate increased genome heterozygosity in the form of new mutations that include alterations in key drug resistance genes. Experimental data imply that defective DNA mismatch repair (MMR) contributes to the development of multidrug resistance by *Plasmodium falciparum* parasites (Castellini et al. 2011). Literature data suggest that the disruption of TgMSH-1, an MSH in *T. gondii*, confers drug resistance.

In conclusion, at present, little is known about MMR and *Toxoplasma gondii* biology. The future of *Toxoplasma* research should reveal more interesting parasite effectors that modulate the mismatch repair.

Repairing double-strand breaks (DSB) is absolutely essential for the survival of obligate intracellular parasite *T. gondii* (Bhattacharyya et al. 2004). DSB are the most lethal of all DNA lesions. If not corrected, it leads to a loss of chromosome segments, threatening cell survival (Hande 2004).

A DSB can be repaired either by homologous recombination (HR) or by nonhomologous recombination (NHEJ) (Walker et al. 2001; Jackson 2002; Christmann et al. 2003; Helleday 2003). During HR-mediated repair of DSB, the sister chromatid is used as a template to copy the missing information into the broken locus. Repair by HR is mediated by Rad51 protein with the help of other members of Rad52, Rad54 and Rad55 epistasis group (Hays et al. 1995).

TgRad51 is the first member of the recombination machinery of *T. gondii* to be characterized. The protein TgRad51 is very important in the recombination machinery of *Toxoplasma gondii* (Achanta et al. 2012). TgRad51 involved in targeted gene disruption has not been characterized yet. The existence of Rad54, Rad50 and Mre11 in *T. gondii* genome suggests that this parasite does possess a functional recombinosome. Interestingly, the apparent lack of Rad52 in *T. gondii* is suggestive of a Rad52 independent recombination mechanism in this pathogen (Achanta et al. 2012).

The NHEJ pathway simply fuses two broken ends with little or no regard for sequence homology. In the NHEJ pathway, Ku70 and Ku80 then bind the DSB, followed by recruitment and activation of DNA–protein kinase (DNA-PK) (Walker et al. 2001). Ku facilitates recruitment of Artemis–DNA-PKcs complex, which processes the ends to prepare them for ligation.

Prokaryotes and lower eukaryotes prefer high-fidelity repair mechanism such as HR, whereas higher eukaryotes show preference toward mutagenic NHEJ pathway. It is knowledge that *T. gondii* prefers NHEJ, which is also used to repair DNA in broken chromosomes, and arbitrarily reinserts targeting DNA segments at incorrect locations. Because the HR machinery is weak in *T. gondii*, targeted gene disruption or tagging of endogenous genes is very less efficient in this parasite (Donald and Roos 1998).

The *Toxoplasma gondii* genome reveals genes encoding putative DNA ligase IV and DNA-dependent protein kinase components of eukaryotic nonhomologous recombination. Experimental results show ku80 to be an essential component of the NHEJ mechanism in *T. gondii* (Fox et al. 2009). Fox et al. showed that in a ku80 knock out background, there is a 300–400-fold increase in targeted gene disruption in *Toxoplasma* parasite (Fox et al. 2009). In the absence of ku80, homologous recombination predominates. Several laboratories have demonstrated enhanced gene targeting in ku80 null *T.* *gondii* (Fox et al. 2009; Huynh and Carruthers 2009). However, as expected, there is no change in gene targeting efficiency in ku80 null parasite lines (Fox et al. 2009). Further research will be necessary to determine precisely how DSB repair is activating in *T. gondii*.

In conclusion, the obtained data suggest that DNA repair mechanisms play an important role in *Toxoplasma gondii* biology. The investigation of DNA repair system during pathogens infection goes on.

## DNA repair contributes to immune system and *T. gondii* infections

Immunological system is very important in response to foreign pathogens such as *Toxoplasma gondii*. Moreover, its maturation and function during human lifespan are dependent on DNA repair machinery.

**Fig. 1:**
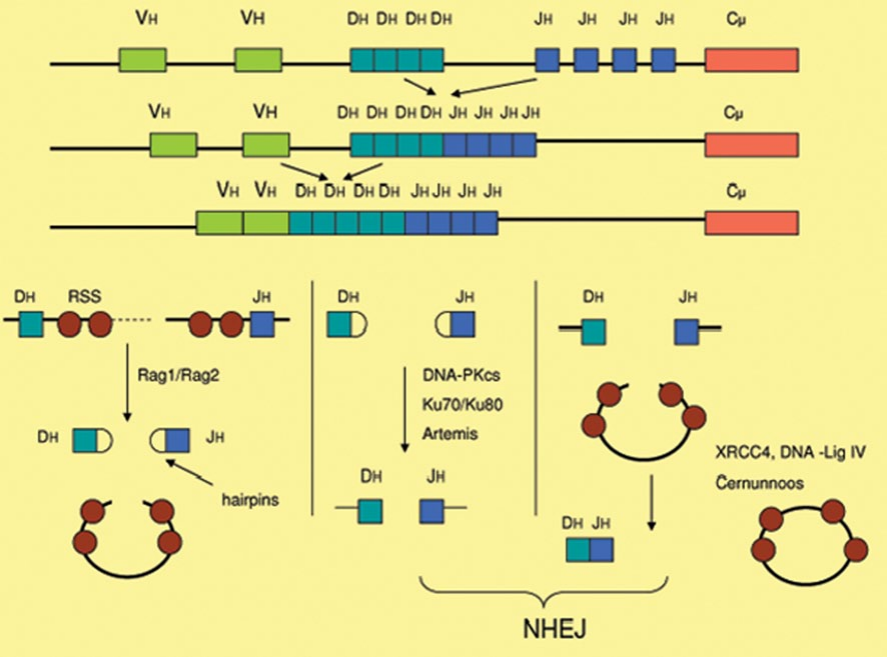
Variable (diversity) joining (V(D)J) encoding gene recombination process. Step 1: The Rag1/2 complex introduces a DNA double-strand break at the border between VH and DH segments and their respective recombination signal sequences (RSS), creating hairpin-sealed coding ends and blunt signal ends. Step 2: Artemis, which is phosphorylated by the Ku/DNA-PK complex, opens the hairpins through its endonuclease activity. Step 3: The XRCC4/Cernunnos/DNA-LigaseIV complex finally seals coding and signal joins

DNA repair system plays a key role during maturation of immune system. Defects in DNA damage response pathways result in block of T lymphocyte differentiation and lead to pathogens and viral infections. B and T lymphocytes respond to invasion of pathogens by specialized antigenic receptor: the T-cell receptor (TCR) and B-cell receptor (BCR). The required diversity of these receptors is ensured by the V(D)J (variable (V), diversity (D) and joining (J) encoding gene) recombination process (Fig. 1). NHEJ pathway is very important in V(D)J recombination (Jain et al. 2009). The rearrangement of V(D)J gene is absolutely essential for many form of TCR and BCR receptors creation. In this process participates a component of the NHEJ repair complex such as DNA-PK complex formed by Ku70 (XRCC6), Ku80 (XRCC5), the DNA-PKcs catalytic subunit (XRCC7, Mu-scid) and the XRCC4/DNA ligase IV (Moshous et al. 2001; Buck et al. 2006). DNA-PK recruits the processing enzyme Artemis, while the XRCC4/DNA ligase IV complex together with Cernunnos terminates the reaction by rejoining the broken DNA ends. Artemis and Cernunnos belong to two novel DNA repair factors (Moshous et al. 2001; Buck et al. 2006).

NHEJ is involved in immunological system maturating, cell–cell and cell–matrix adhesion (Tonegawa 1983). The precise function and activities of these NHEJ factors are very important for immune system development. Defects of NHEJ during receptors maturation may be associated with immunodeficiency and easy pathogen such as *T. gondii* infection. V(D)J recombination defects cause about 20 % of severe combined immunodeficiencies disorders in humans (Revy et al. 2005). Disturbance of the immune system is associated with loss of critical immune functions, such as protection from infection.

## DNA repair gene polymorphism and *T. gondii* infections

DNA repair genes are involved in the control of the genome stability and integrity (Christmann et al. 2003). Table 1 presents the key genes belonging to DNA repair machinery. DNA repair gene is highly polymorphic. The question appears whether genetic polymorphism in DNA repair gene may be correlated with variation in DNA repair efficiency during parasite infection. The presence of the various combined genotype of the single nucleotide polymorphisms of DNA repair genes may be associated with the risk of various diseases. Therefore, the analysis of DNA repair gene polymorphism during pathogen infection may represent an advance in the study of toxoplasmosis pathogenesis.

**Table 1:** The key genes of DNA damage repair pathways

DNA repair mechanism	Gene
Base excision repair (BER)	*APE1*, *APE2*, *APTX*, *DNA2*, *FEN1*, *LIG1*, *LIG3*, *MBD4*, *MPG*, *MUTYH*, *NEIL1*, *NEIL2*, *NEIL3*, *NTHL1*, *SMUG1*, *TDG*, *TDP1*, *UNG*, *XRCC1*, *NUDT1*, *OGG1*, *PARP1*, *PARP2*, *PNKP*, *POLB*, *POLG*
Mismatch mediated repair (MMR)	*EXO1*, *HMBG1*, *LIG1*, *MLH1*, *MLH3*, *MSH2*, *MSH3*, *MSH6*, *PCNA*, *PMS1*, *PMS2*, *POLd*, *RFC*, *RPA*
Nucleotide excision repair (NER)	*CEN2*, *CSA*, *CSB*, *CUL4A*, *DDB1*, *DDB2/XPE*, *ERCC1*, *ERCC4/XPF*, *HR23B*, *LIG1*, *LIG3*, *POL D/E*, *RPA*, *TFIIH*, *XPA*, *XPC*, *XPG*, *XRCC1*
Homology repair (HR)	*ATM*, *ATR*, *BLM*, *BRCA1*, *EME1*, *EXO1* *FANCD/BRCA2*, *FANCF*, *FANCM*, *FANCN*, *GEN1*, *MRE11*, *NBS1*, *Rad50*, *Rad51*, *Rad52*, *Rad54*, *RecQ4*, *RPA*, *WRN*, *XRCC2*, *XRCC3*
Nonhomologous end-joining (NHEJ)	*ARTEMIS*, *ATM*, *ATR*, *DNA*-*PKcs*, *Ku70*, *Ku80*, *LIG4*, *POL4*, *XRCC4*

*Toxoplasma gondii* may be associated with ocular and brain lesions (Furtado et al. 2013; Finsterer and Auer 2012). In the literature, several reports confirm the significance DNA repair genes polymorphism, regarding the risk of ocular and brain diseases (Jacobs and Bracken 2012; Chen et al. 2012; Wibom et al. 2012; Montelli et al. 2011; Kundu et al. 2011; Messaoud et al. 2010, Felini et al. 2007). It is supposed that parasite infection may be correlated with DNA repair genes polymorphism previously implicated in congenital or juvenile onset ocular lesions and brain diseases. The genetics variable has been identified in several DNA repair genes, but the influence of specific genetic variants on repair phenotype and *T. gondii* infection risk has not yet been clarified.

## Conclusion

The pathogenesis of toxoplasmosis remains largely unknown. There is great controversy regarding which factors are responsible for the occurrence or recurrence of toxoplasmosis. DNA repair mechanisms play an important role in *Toxoplasma gondii* biology. The protozoan parasites survival depends on DNA repair systems. In future, understanding of these processes can prevent from parasitic diseases based on not sufficient DNA repair processes.
